# Mouse models of nonalcoholic fatty liver disease (NAFLD): pathomechanisms and pharmacotherapies

**DOI:** 10.7150/ijbs.65044

**Published:** 2022-09-06

**Authors:** Tingyu Fang, Hua Wang, Xiaoyue Pan, Peter J. Little, Suowen Xu, Jianping Weng

**Affiliations:** 1 Department of Endocrinology, Institute of Endocrine and Metabolic Diseases, The First Affiliated Hospital of USTC, Division of Life Sciences and Medicine, Clinical Research Hospital of Chinese Academy of Sciences (Hefei), University of Science and Technology of China, Hefei 230001, China.; 2 Department of Oncology, the First Affiliated Hospital of Anhui Medical University, Hefei, 230022, Anhui, China.; 3 Department of Foundations of Medicine, New York University Long Island School of Medicine, Mineola, New York, NY 11501, USA.; 4 School of Pharmacy, Pharmacy Australia Centre of Excellence, The University of Queensland, Woolloongabba, Queensland, 4102 Australia.

**Keywords:** Drug discovery, mouse model, NAFLD, NASH, pharmacotherapy

## Abstract

The prevalence of non-alcoholic fatty liver disease (NAFLD) increases year by year, and as a consequence, NAFLD has become one of the most prevalent liver diseases worldwide. Unfortunately, no pharmacotherapies for NAFLD have been approved by the United States Food and Drug Administration despite promising pre-clinical benefits; this situation highlights the urgent need to explore new therapeutic targets for NAFLD and for the discovery of effective therapeutic drugs. The mouse is one of the most commonly used models to study human disease and develop novel pharmacotherapies due to its small size, low-cost and ease in genetic engineering. Different mouse models are used to simulate various stages of NAFLD induced by dietary and/or genetic intervention. In this review, we summarize the newly described patho-mechanisms of NAFLD and review the preclinical mouse models of NAFLD (based on the method of induction) and appraises the use of these models in anti-NAFLD drug discovery. This article will provide a useful resource for researchers to select the appropriate model for research based on the research question being addressed.

## 1. Introduction

Nonalcoholic fatty liver disease (NAFLD) is one of the most prevalent liver diseases worldwide. In the coming decades, NAFLD will become the major cause of end-stage liver disease. NAFLD affects both adults and children 1. Between 2016 and 2030, the growth rate of total NAFLD cases is between 0-and 30% 2. A meta-analysis showed that the overall prevalence of NAFLD in Asia was 29.62% 3. The annual incidence of primary liver cancer in the Asian NAFLD population is 1.8‰ 3.

**Figure 1:**
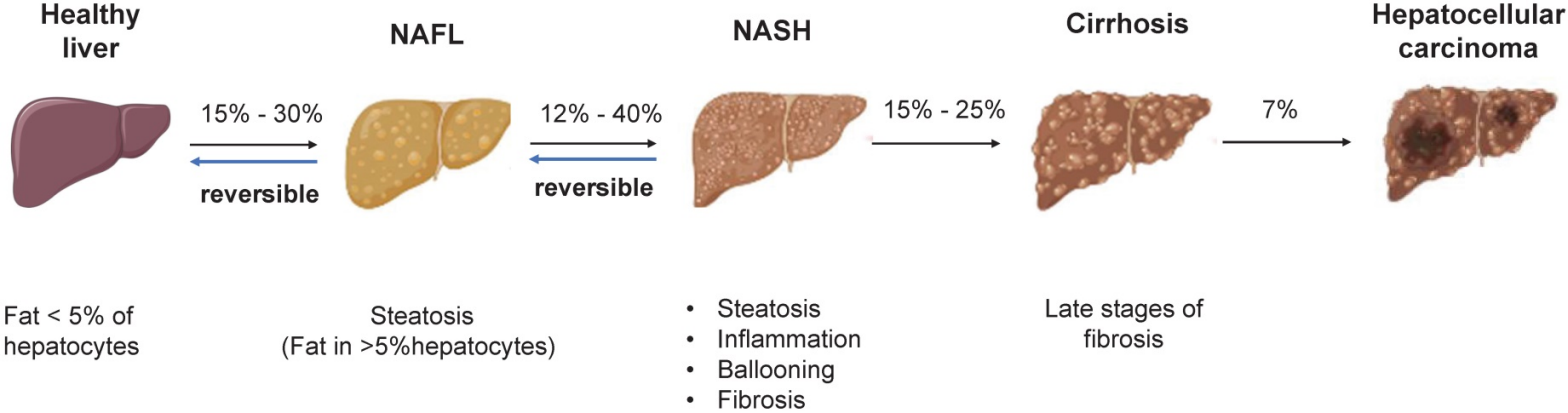
** The pathogenesis of NAFLD.** There is reversibility between normal liver and NAFL, NAFL and NASH. When progressed to NASH, steatosis, inflammation, ballooning degeneration with fibrosis and eventually cirrhosis will occur. The progression of NASH to HCC is an irreversible process and therefore treatment and intervention for NAFLD is suggested to target the reversible stage of disease. Abbreviations: NAFL: Non-alcoholic fatty liver; NAFLD: Non-alcoholic fatty liver disease; NASH: Non-alcoholic steatohepatitis; HCC: hepatocellular carcinoma.

NAFLD is an umbrella term that covers a wide spectrum of liver diseases ranging from nonalcoholic fatty liver (NAFL), liver fibrosis, cirrhosis to hepatocellular carcinoma (HCC) (**Figure 1**) 4. NAFLD begins with the abnormal accumulation of triglycerides in the liver, which triggers lipotoxicity, ER stress and inflammatory response and progresses to cirrhosis even liver cancer. Nonalcoholic steatohepatitis (NASH) is the advanced form of NAFLD characterized by the presence of hepatic steatosis, inflammation, ballooning and/or fibrosis 5. Despite the deepened understanding of the pathogenesis and therapeutic targets of NAFLD/NASH 6, 7, no drug with this indication has been approved by the US-FDA 8. By using mouse models, the study of human diseases and the identification of new therapeutic targets have been significantly facilitated. Therefore, a summary of the existing preclinical models can not only expedite the research of new targets but also contribute to the refinement of animal models. The purpose of this review was to summarize the pathogenesis, preclinical animal models, susceptibility genes and the latest research advances of NAFLD, providing useful information to identify new therapeutic targets and develop new pharmacotherapies.

## 2. Mechanisms of NAFLD/NASH

### 2.1 Role of hepatic steatosis in NAFLD

The lack of approved pharmacotherapies for NAFLD/NASH indicate the need of suitable preclinical animal models which recapitulate human NAFLD pathologies. The liver is an important metabolic organ where *de novo* fatty acid (FA) synthesis occurs. Fatty liver tends to form when excessive FA synthesis causes dysregulated lipid metabolism. Liver fatty mainly derived from hepatic *de novo* lipogenesis (DNL) in human patients 9. DNL related genes include ATP citrate lyase (*Acly*)*, acetyl-CoA carboxylase 1 (Acc1), fatty acid synthase (Fasn),* and so on. Many therapeutic drugs targeting these DNL related genes are currently in different stages of clinical trials 9. Recently, Huh *et al.* 10 found that TANK binding kinase 1 (TBK1) affected the mitochondrial localization of acyl-CoA synthetase long-chain family member 1 (ACSL1) and the oxidation of FA in hepatocytes by binding to ACSL1 during FA oxidation. Besides, the silencing of methylation-controlled J protein (MCJ) in the liver reduces NAFLD pathogenesis by increasing mitochondrial FA oxidation 11. Molecules related to lipid metabolism not only represent therapeutic targets but also serve as potential new biomarkers for NAFLD. For example, the serum level of thrombospondin-1 (TSP1) in patients with fatty liver was increased before treatment, but the serum TSP1 level was decreased after hepatic fat-lowering therapy 12. In addition, microRNAs can regulate hepatic lipid metabolism by regulating the transcriptional activity of key nuclear receptors, such as Liver X receptor alpha (LXRα) and Farnesoid X receptor (FXR) 13. For example, miR-552-3p regulated LXRα and FXR expression by binding to the AGGTCA conserved motif 14. MicroRNA-regulated expression of metabolic receptors offers a promising avenue to search for potential targets for treating fatty liver disease. By feeding mice with a high-fat diet (HFD) to establish a NAFLD model, Zhao *et al.* 15 verified that the inhibition of miR-122 up-regulated Sirt1 expression and the activation of the AMP-activated protein kinase (AMPK) pathway to inhibit adipogenesis. In addition to enzymes, serum proteins, non-coding RNA, and ion channels also affect lipid metabolism. For example, cyclin and CBS domain divalent metal cation transport mediator 4 (CNNM4) is a Mg^2+^ transporter. Knockdown of CNNM4 invoked intracellular Mg^2+^ accumulation, reduced endoplasmic reticulum (ER) stress, and promoted hepatic lipid clearance 16.

### 2.2 Autophagy in NAFLD

In hepatocytes, large lipid droplets (LDs) were divided into small LDs by cytoplasmic lipolysis, followed by lipophagy-dependent degradation 17.

A recent study has demonstrated that liver-specific knockdown of acyl-coenzyme A (CoA) oxidase 1 (Acox1) protected mice from starvation- or HFD-induced hepatic steatosis by inducing autophagic degradation of LD 18. Acox1 deficiency in the liver reduced the total cytoplasmic acetyl-CoA level, which led to decreased lysosomal localization of mammalian target of rapamycin (mTOR) 19. Acox1 attenuates mice NAFLD via different mechanisms illustrating that Acox1 is a potential target for NAFLD.

Dynamin-related GTPases for division (Drp1) phosphorylation activated autophagy. When Drp1 was abrogated in the liver, insufficient splitting of mitochondria caused mitophagy and p62 (autophagy ligase protein) upregulation to recruit two subunits of the E3 ligase complex, Keap1 and Rbx1, to promote ubiquitination of mitochondrial proteins 20. This is similar to diet-induced NAFLD in which mitochondria size expands and accumulates mitochondrial autophagic intermediates.

### 2.3 ER stress in NAFLD

Hyperlipidemia, inflammation, virus infection, and drugs can disrupt metabolic homeostasis in the ER of hepatocytes 21. ER controls liver protein and lipid homeostasis through unfolded protein response (UPR) 22. UPR was originally perceived as a normal physiological mechanism to tackle aberrant protein turnover, but it subsequently transformed into chronic stress and impaired hepatocyte function to stimulate inflammation and cell death in NAFLD.

In a recent study, Liu *et al.* 23 have identified a novel role of the Xbp1-Foxa3- Period1/Srebp1c signaling axis in NAFLD. Mechanistic investigations showed that Foxa3 directly regulated Period1 transcription in mice, which in turn promoted the expression of lipogenic genes and led to NAFLD development 23.

In addition to Foxa3, some microRNAs and circular RNAs (circRNAs) serve as the bridge molecule between ER stress and NAFLD. For example, overexpression of miR-26a mitigated palmitate-triggered ER stress and lipid accumulation in human hepatoma cells and murine primary hepatocytes 24. Targeted delivery of the newly-identified circRNA-SCAR inhibited the generation of mitochondrial reactive oxygen species (ROS) and the proinflammatory phenotype of NASH 25. These findings provide further evidence supporting that mitochondrial ROS mediated the proinflammatory phenotype in hepatocytes after exposure to lipotoxic stimuli.

### 2.4 Ferroptosis in NAFLD

Deregulated iron metabolism also accelerates the progression of NAFL to NASH. A clinical study has shown that hepatic iron loading affects hepatic fat content in dialysis patients 26. Researchers also found that ferroptosis in an ethionine-supplemented diet (CDE) model was involved in the initial necrotic cell death and induced steatohepatitis 27. In contrast, ferroptosis inhibitors can slow the progression of NAFLD 28, 29. Sorafenib, antifibrotic drug, trigger ferroptosis via HIF-1αonly in hepatic stellate cells (HSCs) but not hepatocytes 30. Ferroptosis is still a less studied pathogenic mechanism, and further research is warranted to discover novel ferroptosis-related targets in NAFLD.

### 2.5 Inflammasome activation in NAFLD

In methionine- and choline-deficient diet (MCD) and HFD induced model of NAFLD, mRNA level of NLRP3 was higher than control group 31. A recent study has demonstrated that palmitic acid (PA) promoted the activation of NLRP3 inflammasome in hepatocytes, and NLRP3 inflammasome activation was inhibited by adiponectin through the AMPK/JNK/ERK1/2-NFκB/ROS signaling pathway 32. NLRP3 inflammasome amplified inflammation and triggered pyroptosis. Hepatocyte pyroptosis and the release of inflammatory components are new mechanisms for the development of liver injury and liver fibrosis 33.

### 2.6 Role of immune regulators in NAFLD

Emerging evidence has shown that immune regulators play essential roles in NAFLD. For example, mitochondrial antiviral signaling protein (MAVS) is a part of innate immunity and antiviral response 34. In NASH, mitochondrial damage extends to MAVS, resulting in a decrease in the induction of type I interferons 35. MAVS is the downstream adaptor protein of RIG-I/ Melanoma differentiation-related gene 5 (MDA5, also known as Ifih1). After binding, it can induce the production of type 1 interferon and activate NF-κB, IRF-3 and other pathways. MDA5, as the target of apoptosis signal-related kinase 1 (ASK1), is an important inhibitor of NASH induced by an HFD/ high-fat and high-cholesterol diet (HFHC) 36. Knolle *et al.* 37 reported the mechanism of T-cell response to NASH-associated liver injury in a CD-HFD mouse model. Forkhead box O1 (FOXO1) was downregulated and C-X-C motif chemokine ligand 6 was upregulated after IL-15 induction in T cells, leaving the CXCR6^+^CD8 T cell susceptible to metabolic stimuli. ATP released triggered rapid Fas ligand (FasL) upregulation and self-attack in CD8 T cells, causing liver injury. Meanwhile, Pfister *et al.* 38 used the same animal model to discover the involvement of CD8 T cells in the induction of HCC due to NAFLD. Patients with advanced HCC found that immunotherapy was ineffective in improving HCC due to NAFLD. This condition is probably due to unconventional activation of T cells and auto-attack, resulting in compromised immune system function.

### 2.7 Extracellular vesicles (EVs) in the development of NAFLD

It is well established that almost all cell types in the liver can secrete exosomes, such as hepatocytes, macrophages, neutrophils and so on 39. Other metabolic organs, such as adipose tissues, can also secrete exosomes. The cargos transported by exosomes include lipids, proteins and nucleic acid 40. The exchange of EVs between neutrophils and liver cells can improve or promote NASH dependent on the transported cargo. The increment of miR-223 in liver cells is due to the preferential uptake of miR-223-rich EVs derived from neutrophils. miR-223 improve NASH in the mouse model by upregulating the expression of low-density lipoprotein receptor (LDLR) 41. Overexpression of CXCL1 and aldo-keto-reductase 1B7 (Akr1b7) can also induce NASH 42, 43. Noteworthy, deletion of Akr1b7 (from adipocyte-secreted exosome which generated by ER stress) protected mouse liver from NASH induced by feeding with HFD and MCD. *In vivo*, CXCL1 overexpression in HFD-fed mice increased the mtDNA content in EV. Lysophosphatidylcholine (LPC), cholesterol and other lipid mediators induced hepatocytes to secrete exosomes which contain integrins and microRNAs. EVs from hepatocytes undergoing lipotoxic stress are rich in integrin β_1_ (ITGβ_1_) and microRNA 192-5p 44, 45. The adhesion of monocytes to liver sinusoidal endothelial cells (LSECs) mainly depends on ITGβ_1_, while miR-192-5p plays a role in the activation of proinflammatory macrophages and NAFLD pathogenesis by regulating Rictor/Akt/FoxO1 signaling.

### 2.8 Gut microbiota in NAFLD

The intestinal mucosa of healthy people is intact. This helps maintain the integrity of the intestinal flora composition and resists bacterial invasion 46. In recent years, emerging evidence has shown that the gut microbiota regulates the pathogenesis of NAFLD. Mice after a short-term of HFD feeding (1 week), experience gut vascular barrier (GVB) damage and bacterial translocation into the liver 47. In turn, transplanting fecal bacteria from these HFD-fed mice to specific pathogen-free recipients also caused GVB damage 47. The composition of the gut microbiota changes with the pathogenesis of NAFLD 48. Gut microbiota and its metabolites promote the formation of an immunosuppressive environment 34.

### 2.9 Fibrogenesis and NAFLD

Fibrosis is the late stage of NAFLD, with the activation of HSCs being the core cellular mechanism. After activation, HSC express high levels of extracellular matrix proteins leading to the formation of fibrosis. Farnesoid X receptor (FXR) is a bile acid receptor. It regulates bile acid secretion, which can down-regulate the expression of crucial lipogenesis-associated genes in the liver and ultimately reduce hepatic lipid content 49. A previous study has shown that SUMOylation inhibitors can increase the inhibitory effect of prophylactic obeticholic acid (OCA, an FXR agonist) on HSC activation and fibrosis 50. Acetyl-CoA carboxylase (ACC) is an important target for regulating DNL. Inhibition of ACC reduces the lipotoxicity of liver cells and inhibits the activation of HSCs 51.

## 3. Mouse models of NAFLD

“Multiple-hit” hypothesis is a well-accepted one for NASH development 52. To enhance our understanding of NAFLD's pathomechanisms, the use of animal models is very helpful. Animal models are also helpful for exploring new therapeutic modalities. An ideal animal model should be able to reproduce the complex pathomechanisms of human disease pathology with high fidelity. This section summarizes the most commonly used animal models in the study of NAFLD, highlighting the major advantages and disadvantages of each model (**Table 1**).

### 3.1 Dietary model

#### 3.1.1 High-fat diet (HFD)

There are different types of HFD in terms of nutrient composition, and this difference may also be caused by different sources (animal or vegetable) and amounts of fat. Conventional HFD usually contains 60 kcal% or 45 kcal% fat and mice fed with HFD will develop obesity, insulin resistance (IR) and hepatic inflammation after 16 weeks of feeding 53. Mice fed HFD for 1 week can develop diet-induced ecological dysbiosis, leading to bacterial translocation to the liver 47. Therefore, HFD is an appropriate choice to study the effect of gut microbiota on the development of NAFLD. The HFD-fed mouse model can be used to verify the mechanism of action of some drugs such as water extract of shepherds purse (WESP) 54 and ursolic acid in alleviating NAFLD development 55. In mice, different strains and genders show different sensitivities to HFD. Therefore, variables in selecting this model include dietary fat content, duration and strains. The model also displays similar features to human NAFLD/NASH development and is widely used in metabolic research, but the pathological outcome was less severe than human NASH, particularly the fibrogenesis process 56.

#### 3.1.2 High-fat high-fructose diet (HFFD)

The second frequently-used model is HFFD model by adding 10% fructose to the HFD. However, the extent of steatosis, inflammation and fibrosis were ameliorated in fructokinase knockout mice 57. Fats can come from animal or plant sources 58, 59. Excessive fat and fructose intake cause IR, inflammation and ER stress through different mechanisms, such as free fatty acid (FFA)-derived lipotoxicity, and the expression of proinflammatory cytokines and chemokines 60-62. Accordingly, the HFFD model is a good choice when studying the role of ER stress and lipid steatosis in NAFLD. A recent study has compared the differential effect of types of diets on NAFLD in mice with that in human patients, and conclude that HFFD model best recapitulates the human phenotype of NAFLD 63.

#### 3.1.3 Methionine- and choline-deficient diet (MCD)

MCD is rich in sucrose, which provides the right amount of fat but lacks methionine and choline. Methionine deficiency can lead to the blockade of protein synthesis in the body 64. Choline is a strong organic base, a constituent of lecithin and a precursor of acetylcholine. Recently, the MCD model has been used to study the role of gut microbiota and immune response in NAFLD. The activation of macrophages involved the interaction of hypoxia-inducible factors 1 alpha (HIF-1α) and autophagy, which promoted the proinflammatory overactivation of in a preclinical model induced by MCD 65. Additionally, in kupffer cells residing in the liver, the stimulator of IFN genes (such as the stimulator of interferon genes (STING) induced inflammation through the IRF and NF-κB pathway 66. Symbiotic microbiota was found to have hepatoprotective effects in MCD-induced steatohepatitis. Unfortunately, the composition of the intestinal flora of MCD diet-fed mice is not similar to that of humans 67. In addition, MCD model is a commonly used model for studying ferroptosis. MCD-fed mice have iron accumulation in the liver and serum. Ferroptosis inhibitors can reduce liver damage and liver fibrosis caused by MCD feeding 28, while ferroptosis inducers increased lipoxygenase and apoptosis-inducing factors and affect lipid oxidation and other types of cell death 29.

#### 3.1.4 Choline-deficient L-amino-defined diet (CDAA)

Dietary choline deficiency, L-amino acid defined diet (CDAA) is another popular model for pharmacological and genetic research of NAFLD 68, 69. Like MCD, CDAA is a choline-deficient diet in which sulfhydryl-containing amino acids are less restricted and ample energy is supplied by the HFD. The methionine level in the semisynthetic CDAA diet was normal or only moderately reduced, while the protein content in the formula was replaced by an L-amino acid mixture 70. After 12 weeks of feeding with CDAA, steatohepatitis developed with fibrosis and an approximately three-fold increment in liver collagen level was observed. After 21 weeks of feeding, the fibrosis progresses to a moderate stage. In addition, CDAA model showed liver cancer-associated fibrosis, thereby accelerating the study of disease progression from NASH to HCC 71.

#### 3.1.5 Choline-deficient, L-amino acid-defined, high-fat diet (CDAHFD)

Compared with the above models, choline-deficient, L-amino acid-defined, high-fat diet (CDAHFD) is another widely-used model in NAFLD/NASH studies. This model simulates human NAFLD pathology much more closely 72. The most commonly used formula for CDAHFD is a high-fat, choline-deficient diet, including 0.1% methionine and 45% fat 73. After 6 weeks of feeding, the CDAHFD model exhibited hepatic steatosis, liver damage, and inflammation 73. CDAHFD feeding for 6 weeks triggers liver fibrosis evidenced by picosirus red staining 74. Furthermore, C57BL/6J mice fed with CDAHFD can develop steatohepatitis without affecting body weight 74. Interesting features of this model also include i) CDAHFD can induce steatohepatitis within one week, together with mitochondrial dysfunction and severe oxidative stress, but without fibrosis. ii) The content of methionine in the formula determines whether or not the mouse can become obese 75, 76. This is a unique feature that other models do not have, and it simulates some of the characteristics of human NAFLD. The CDAHFD model has significant applications in studying the role of novel therapeutic targets (such as TSP-1, transient receptor potential canonical (TRPC) 77, 78, traditional Chinese medicine (such as nobiletin, astaxanthin) 79, 80 or western medicine (such as metformin and PPAR agonist) in NAFLD/NASH development 74, 81.

#### 3.1.6 High-fat high-cholesterol diet (HFHC)

Dietary cholesterol is an important factor associated with the progression of steatohepatitis and liver inflammation in mouse models and humans 82. Wang *et al.* found that hepatocyte cholesterol activated TAZ (a transcriptional regulator that promotes fibrosis) leading NASH and liver fibrosis. Mice fed with 30% fat, 1.25% cholesterol, and 0.5% cholate have a ballooning-like expansion of hepatocytes, an essential characteristic of human NASH 83.

The high-fat and high-cholesterol diet (HFHC) model was widely used in NAFLD/NASH research 84. This model can be used for drug evaluation and probing molecular mechanisms of NAFLD. For example, cordycepin, a potentially new natural AMPK activator for treating NASH, relieves hepatic steatosis, inflammation, liver injury and fibrosis in the NASH mouse model established by feeding mice with HFHC diet 85. In addition, cholesterol diets induced modification in intestinal microflora and metabolites to promote NAFLD development 86. Thus, HFHC feeding is also used to study the influence of intestinal microorganisms on NAFLD 86, 87.

#### 3.1.7 High-fat, fructose, and cholesterol model (HFFC)

The high-fat, high-fructose, and high-cholesterol diet (HFFC) model is frequently used in establishing animal models of metabolic syndrome. HFFC diet usually contains 40% fat, 20% fructose, and 2% cholesterol, which was previously known as the Amylin Liver NASH (AMLN) diet 88. After 34-36 weeks, liver TG but not serum TG has increased in mice 89. Liver injury was observed evidenced by elevated levels of serum AST/ALT 89. Compared to the control group, expression of fibrosis-related genes, such as *collagen 1α1 (Col1a1), α-SMA (Acta2), and lysyl oxidase-like 2 (Loxl2)* were elevated and prominent collagen accumulation was detected by picosirius red staining 90. Recently, Song *et al.* found 25-hydroxylanosterol (25-HL) has preventive and curative effects on NASH mice fed with HFFC diet 91. Mechanistically, 25-HL binds to insulin-induced gene, promoting SCAR-SREBP retention in ER, thereby reducing the level of cholesterol and TG. Another unique feature of this model is that HFFC diet-treated Ldlr^-/-^ mice simultaneously induced the development NASH and atherosclerosis 91. Therefore, this model has tremendous utility in developing new therapeutic targets as well as new drugs which ameliorate NASH and atherosclerosis in light of the fact that most NAFLD patients die from extrahepatic complications, such as atherosclerosis.

#### 3.1.8 High-fat diet plus carbon tetrachloride (CCl_4_) induction

Carbon tetrachloride (CCl_4_) is a hepatotoxic chemical which causes liver injury, liver fibrosis and cirrhosis in experimental animals. Repeated administration of CCl_4_ to HFD-fed obese mice successfully induced chronic oxidative stress, triggered inflammation and led to liver fibrosis 92. Notably, under feeding with Western diet (WD) supplemented with 5% fructose (WDF), CCl_4_ reduced the induction time and aggravated liver fibrosis in FATZO mice 93. In comparison to CDAA- treated mice, additional treatment with a single dose of CCl_4_ resulted in not merely hepatic lipid deposition but also peri-hepatocellular fibrosis 94. HFFC diet combined with a low-dose of CCl_4_ injection intraperitoneally to establish a murine NASH model with extensive fibrosis and rapid HCC progression 95. The advantages of this model are i) generalization from simple steatosis to inflammation, fibrosis and hepatic cancer. ii) simplicity and reproducibility of the model facilitate the study of disease pathogenesis and the tests of new treatments. Similar model has been established in rats by HFFC diet and CCl_4_ treatment 96. This is a preclinical model of moderate and advanced NASH that mimics human disease and exhibits almost all the characteristics of advanced human NASH after 10 weeks and cirrhotic NASH after 24 weeks. However, very few evidence exists as to whether NASH induced by this model can be regressed by diet switch.

#### 3.1.9 STAM model

Like HFFC plus CCl_4_ model, STAM model also induced NASH and even HCC by combining diet and chemical treatment. Two-day-old nascent C57BL/6J male mice were injected low-dose streptozotocin (STZ) (200 μg per mouse) 97. Mice were fed a high-fat/high-calorie diet after 4 weeks 98. Those mice developed liver steatosis at 7 weeks, reached cirrhosis at 12 weeks, and developed HCC within 20 weeks 98. Steatohepatitis, inflammation, ballooning and fibrosis were observed in those mice 99. Pathological analysis revealed that treated mice have mild steatosis, more severe inflammation and 62.5% of mice have severe ballooning 98. However, the limitation of STAM model lies in the administration of STZ, which does not mimic human NAFLD conditions 100.

### 3.2 Genetic models

#### 3.2.1 CXCL1 plus HFD mouse model

Up-regulation of chemokine production by liver neutrophils is characteristic of human NASH. HFD feeding alone is difficult to trigger the development of NASH in mice. However, overexpression of the chemokine CXCL2 in the liver can induce NASH in mice fed a HFD 42. When CXCL1 is overexpressed in the livers of HFD-fed mice, infiltrating and activated neutrophils produce a high level of reactive oxygen species that contribute to liver injury under chronic inflammatory conditions in NASH. CXCL1 overexpression led to p38α activation that induces the cleavage of caspase-3, C/EBP Homologous Protein (CHOP) expression, and BCL2 phosphorylation, thereby exacerbating hepatocyte death 101. However, mice fed a HFD only showed weak p38α activation, which upregulated genes involved in fatty acid β-oxidation that may act to compensate for hepatocyte lipid accumulation 101. The new NASH model established by hepatic overexpression of CXCL1plus HFD feeding also mimics human NASH pathology. High level of CXCL1 compensated the effects caused by other factors to a certain extent. Together, based on the HFD model, the liver-specific overexpression of the key factors in driving NASH progression can be a new means to establish the NASH model.

#### 3.2.2 microRNA deficiency-induced mouse model

MicroRNA has been increasingly regarded as a critical regulator of metabolism and inflammation in the liver. For example, microRNA 122 (miR-122) is a liver -enriched miRNA, accounting for 70% of the total liver microRNA 102. Long-term inhibition of miR-122a in the liver reproduced human liver pathology, but the short-term decrease of miR-122a expression is beneficial 103. In another study using the NASH mouse model and serum samples from NASH patients, the level of miR-223 is up-regulated 104, 105. MiR-223-KO mice developed NASH after feeding a HFD or MCD for 3 months 106. Genetic deletion of miR-223 induces a full spectrum of NAFLD in long-term HFD-fed mice. In terms of mechanism, miR-223 prevents the progression of steatosis to NASH by inhibiting the expression of Cxcl10 and Taz in the liver 106.

#### 3.2.3 ob/ob mouse model

Obesity is a significant risk factor for NAFLD. The most prevalent genetic models of obesity are leptin (ob/ob) and leptin receptor (db/db) deficient mice 107. Ob/ob mice have spontaneous mutations in the OB gene (encoding leptin), resulting in leptin deficiency. When fed a normal chow diet, ob/ob mice are hyperphagic, inactive, and extremely obese and have many features such as IR, hyperinsulinemia, and spontaneous liver steatosis similar to human NAFLD 62, 108, 109. An experiment conducted to explore different NASH mouse models suggests that ob/ob mice fed fast-food diet or HFD showed metabolic, histological and transcriptome dysfunction similar to human NASH 110.

#### 3.2.4 db/db mouse model

Db/db mice deficient in the DB gene (encoding leptin receptor), are leptin resistant with or without hyperleptinemia 111. In terms of fat accumulation and adipose inflammation, db/db fed with chow diet is comparable to C57BL/6J mice fed with HFD. Db/db mice can show the characteristics of human type 2 diabetes 112. NAFLD pathology in db/db mice can be accelerated by feeding mice with a western-type diet 113.

#### 3.2.5 Foz/foz mouse model

Foz/foz mice carry a mutation in Alström syndrome protein 1 (Alms1) which leads to primary ciliary dysfunction. Patients with Alström syndrome are more likely to develop childhood type 2 diabetes mellitus (T2DM) than patients with other syndromes, suggesting that ALMS1 may be of significance in the cell function and/or peripheral insulin signaling pathway 114. A study has shown increased obesity, glucose intolerance and accelerated NASH pathology in foz/foz mice after 6 weeks of HFD feeding 115. Foz/foz mice fed with HFD developed NASH with fibrosis after 12 weeks, which was alleviated when the diet was switched to a normal diet. As with other genetically NAFLD mouse models, the foz/foz model requires challenge with HFD to induce the occurrence of late-stage of NAFLD phenotype.

Because the pathogenesis of fatty liver is diverse and heterogenous, the mouse models available have similar pathological features but not necessarily the same processes as we see in the clinic. The ARRIVE guideline which was established by the editors of major biomedical journals provides some principles for selecting the right type of animal models in scientific research 94.

## 4. Use of mouse models to study the mechanisms of NAFLD

The establishment of these preclinical animal models can also be used to investigate the impact of disease-relevant targets on the development of NAFLD and the search for potential therapeutic agents (**Table 2**).

### 4.1. PNPLA3

Patatin-like phospholipase domain 3 (PNPLA3) is located on chromosome 22 which encodes protein adiponutrin. Point mutation of PNPLA3 (I148M) promoted steatosis by inhibiting lipid droplets (LDs) degradation via cofactor CGI-58 116. A study by Yang *et al*. 117 revealed a stronger binding affinity of PNPLA3 with adipose triglyceride lipase (ATGL) (compared to CGI-58), which reduced lipid degradation. However, the PNPLA3^I148M^ mutant has a stronger binding affinity to CGI-58 than ATGL and PNPLA2 by suppressing its ubiquitination and protein degradation 116-118. Studies have shown that PNPLA3^I148M^ mutant, but not wild-type PNPLA3, shows fatty liver phenotype and other metabolic characteristics similar to human patients 119. Based on this, Lindén *et al*. 120 constructed a mouse model of human PNPLA3^I148M^ knock-in mutation. The injection of liver-targeted GalNAC_-_conjugated antisense oligonucleotides (ASO) that mediates Pnpla3 silencing improved all the characteristics of NAFLD in this mouse model, including liver fibrosis 120. In addition to PNPLA3^148M^, the PNPLA3 rs738409 variant is associated with the early stage of NAFLD diagnosis 121.

### 4.2. TM6SF2

Genetic mutation of transmembrane 6 superfamily member 2 **(**TM6SF2) plays a role in liver diseases. Emopamil binding protein (EBP) is a cholesterol biosynthetic enzyme. TM6SF2 was confirmed to be homologous to EBP and shared the same domain structure 122. In addition, ATP-binding cassette sub-family G member 5 (ABCG5) promoted reverse cholesterol transport in the liver of TM6SF2 knockout mice 123. TM6SF2 also affects the levels of VLDL and ApoB. Kozlitina *et al*. 124 simulated the effects of TM6SF2-167 Lys mutation on hepatic triglyceride content and blood lipids in high sucrose diets model. Tm6sf2 increased triglyceride (TG) content in the liver of mice and decreased the secretion of VLDL. Studies performed in TM6SF2 depleted or TM6SF2 knockout mice suggest that TM6SF2 reduced ApoB levels and led to lipid accumulation in the liver 125.

### 4.3. HSD17B13

Hydroxysteroid 17-beta dehydrogenase 13 (HSD17B13) was initially named as SCDR9 and was cloned from a human liver cDNA library for the first time in 2007 126. Most of the HSD17B family members are involved in regulating the biological activity of sex hormones, FA metabolism, cholesterol biosynthesis and the production of bile acid (BA) 127. Human genetic research has shown that the splice variant of HSD17B13 (rs72613567: TA) prevented NAFLD development 128. In addition, a meta-analysis has demonstrated that SNP rs72613567 reduced the severity of NAFLD in humans 129. The above studies have indicated that mutating or inactivating HSD17B13 in humans can resist NAFLD. Ma *et al*. 130 used a murine model to study the *in vivo* function of HSD17B13. The authors compared Hsd17b13 knockout (KO) mice and wild-type (WT) littermate controls fed with a regular diet, HFD, WD, or NIAAA alcohol exposure models (a mouse model of chronic and binge ethanol feeding). Compared with WT mice, KO mice showed higher body weight and liver mass; furthermore, KO mice on an obese diet had larger lipid droplet size. Additionally, HSD17B13 KO mice fed a soy-free, natural-ingredient diet showed steatosis and inflammation 131.

### 4.4. TAZ

Higher transcriptional activity of TAZ (also known as WW-domain containing transcriptional regulator 1, WWTR1) was observed in human and mouse NASH hepatocytes compared with normal or simple steatotic hepatocytes. Back in 2016, Wang *et al*. 132 found that TAZ depletion prevented or reversed liver inflammation, hepatocyte death and fibrosis, but not steatosis in mice fed a high-fat and high- fructose diet. After feeding mice with NASH diet for 9 weeks, targeted delivery of siTAZ to the liver can reduce the expression of inflammation and fibrosis associated marker genes, *Col1a1, Col3a1 and α-SMA* 133. These findings indicate that TAZ can be a therapeutic target for NASH.

In addition, hepatocyte-specific TAZ deletion downregulates p62 expression in NASH models and plays a role in inflammation and liver injury 134. Of translational relevance, rosemary acid (RA), a natural product isolated from medicinal plants was reported to slow down the pathogenesis of NAFLD by downregulating TAZ and upregulating PPARγ and PGC-1α in HFD-induced NAFLD model 135.

## 5. Use of mouse models to study the pharmacological actions of drug candidates in NAFLD clinical trials

### 5.1 Selonsertib (GS-4997)

The activity of ASK1 is increased in mouse and human NAFLD samples and is finely tuned by extracellular and intracellular signals. Selonsertib (SEL) is an inhibitor of ASK1. SEL inhibited NASH in established NASH models, such as MCD model and CDAHFD model. SEL (30 mg/kg, q.d.) reduced collagen area and inflammation but did not affect steatosis in MCD and CDAHFD models 136. The improvement of inflammation and fibrosis by SEL was also verified in CCl_4_ plus MCD mouse model 136.

STELLAR-4 is a phase III (NCT03053063), randomized, double-blind, placebo-controlled study to evaluate the safety and effectiveness of SEL in patients with compensated liver cirrhosis (F4) caused by NASH. In short, although SEL improved steatosis and fibrosis in diet-induced mouse models, it did not improve liver fibrosis in patients with advanced NAFLD.

### 5.2 Saroglitazar

Saroglitazar (SAR) is a dual PPARα/γ agonist. In CDAHFD-induced NASH mouse model, SAR reduced liver steatosis and inflammation and reduced the levels of biomarkers of liver injury and inflammation 137. SAR outperforms pioglitazone in HFFC model despite no significant influence on liver and body weight was observed 138. The authors observed that SAR (3 mg/kg, oral administration) treatment for 12 weeks could reverse NASH development. SAR (3 mg/kg, oral) also mitigate the expression of pro-inflammatory genes (*Tnfα*, *Mcp-1*), fibrotic genes (*α-SMA*, *Col1a1 and Ctgf*), and collagen area in CDAHFD induced NASH model 137. Notably, the decrement of fibrosis-related expression wasn't observed in fenofibrate (100 mg/kg) or pioglitazone (30 mg/kg).

In March 2020, the Drug Control Center of India approved SAR's new drug application, which is the world's first drug for the treatment of non-cirrhotic NASH. The drug met the primary and secondary endpoints in the phase II clinical trial (NCT03863574). However, the sample size of this trial is small, including only 16 participants.

### 5.3 Obeticholic acid (OCA)

Farnesol X receptor (FXR) is highly expressed in the liver and plays a role in enterohepatic circulation and BA synthesis. OCA is a FXR agonist. In a NASH model, OCA had higher efficacy and longer half-life than the dual FXR/TGR5 agonist INT-767 139. Diet-induced dysbiosis occurred after one week of HFD feeding 47. In one study, OCA treatment prevented the gut vascular barrier (GVB) integrity. Besides, melanocortin 4 receptor-deficient (MC4R-KO) mice can develop NASH when fed a HFD. This model developed obesity and IR and was used to validate the anti-fibrotic effects of OCA 140. OCA also alleviated inflammation and the progression of fibrosis in HFD-fed Ldlr^-/-^ Leiden mice by reducing collagen deposition and limiting *de novo* lipogenesis 141. Steatosis and fibrosis occurred in FATZO mice when fed a WD supplemented with fructose (WDF) 142. FATZO mice were used to evaluate the effect of OCA on NASH progression. In addition, in another model with ob/ob mice fed an AMLN diet for 15 weeks, the effect of OCA and elafibranor (ELA) in combination on liver histological changes was evaluated. The results showed that OCA reduced liver weight in mice compared to ELA, reflecting an improvement in steatosis by OCA.

In one clinical trial (NCT01265498), patients were randomly assigned to receive OCA treatment (n=141), or placebo treatment (n=142). OCA improved the histopathological characteristics of NASH 143. After receiving OCA treatment, some patients have increased harmful cholesterol in their bodies. While, the FXR target reduced circulating cholesterol by inducing anti-cholesterol transport proteins such as scavenger receptor B1 (SCARB1/SR-B1) and the ATP binding cassette G8 transporter in mice model 144, 145. These differences were also confirmed in human hepatocyte chimeric mice 146.

### 5.4 Liraglutide

Glucagon-like peptide 1 (GLP-1) is an insulin-stimulating hormone produced in L cells and secreted after food ingestion 147. Liraglutide is an analog of GLP-1, which can improve the function of pancreatic β-cells and reduce the body weight of obese patients 148.

Interestingly, liraglutide has been proven to be effective in mouse models of NASH. For example, Moreira et al. 149 evaluated the effects of liraglutide on obesity and NAFLD in two obese mouse models (the ob/ob mice and the HFD mice). Liraglutide improved NAFLD pathologies in mice with induced diabetes by modulating inflammatory signaling pathways 150. In addition, liraglutide prevented the accumulation of ceramide/sphingomyelin in MCD-fed mouse liver 151. Similarly, in db/db mice, ob/ob mice and HFD mouse models, liraglutide has appreciable therapeutic effects on NAFLD. The underlying mechanism is related to the alteration of the composition and diversity of intestinal microbes 149, 152. The therapeutic effects of liraglutide exhibited in the diet-induced obesity (DIO)-NASH and ob/ob-NASH mouse models are translated into observed clinical benefits in NASH clinical trials 153.

### 5.5 Cenicriviroc

Cenicriviroc (CVC) is a C-C chemokine receptor type 2 (CCR2) and type 5 (CCR5) dual antagonist that inhibits liver fibrosis 154. In high-fat, high-fructose models, CVC (0.1% wt/wt, a target dose of 100 mg/kg/day, 8 weeks) administration with diet inhibited liver inflammation and fibrogenesis, facilitating the development of NASH and fibrosis 155. According to the phase IIb clinical trial (CENTAUR study, NCT02217475), fibrosis was improved after 1 year of CVC treatment compared to placebo treatment 156. Thus, CVC has shown anti-fibrosis ability both in preclinical models and clinical trials.

The full list of drugs in clinical stage of development is summarized in **Table 3**.

## 6. Concluding remarks and future perspectives

The pathogenesis of NAFLD involves rather a complex interaction among different mechanisms, based on which the “multiple-hit” theory is the predominant theory. Mouse models are widely used because they are inexpensive, and convenient for genetic engineering. However, they also face the shortcomings due to the inability to fully replicate the characteristics of human NAFLD/NASH. As discussed, each mouse model has its own advantages or disadvantages, and continued efforts to establish new mouse models that can better recapitulate the process and mechanism of human NAFLD are urgently needed.

At the same time, the development of new technologies, such as 3-D organoid derived from human induced pluripotent stem cells (hiPSC), combined with animal models, will greatly accelerate the translation from basic science to clinical discoveries since hepatocytes are terminally differentiated. For example, Wang* et al.* 157 established a human NAFLD-like model based on a liver organoid chip system derived from hiPSC. This system can phenocopy the pathological features of NAFLD in liver organoids by prolonged exposure to FFA in perfused 3D-cultures. Likewise, Jarai *et al.* 158 co-cultured primary human hepatocytes with HSCs, Kupffer cells and liver sinusoid endothelial cells to produce a 3D human liver microtissue (3D-hLMT) system with NASH-like features. Due to the negative impact of this disease on human health and economic burden, NAFLD/NASH will become a high-priority area for future research worldwide.

## Figures and Tables

**Figure 2:**
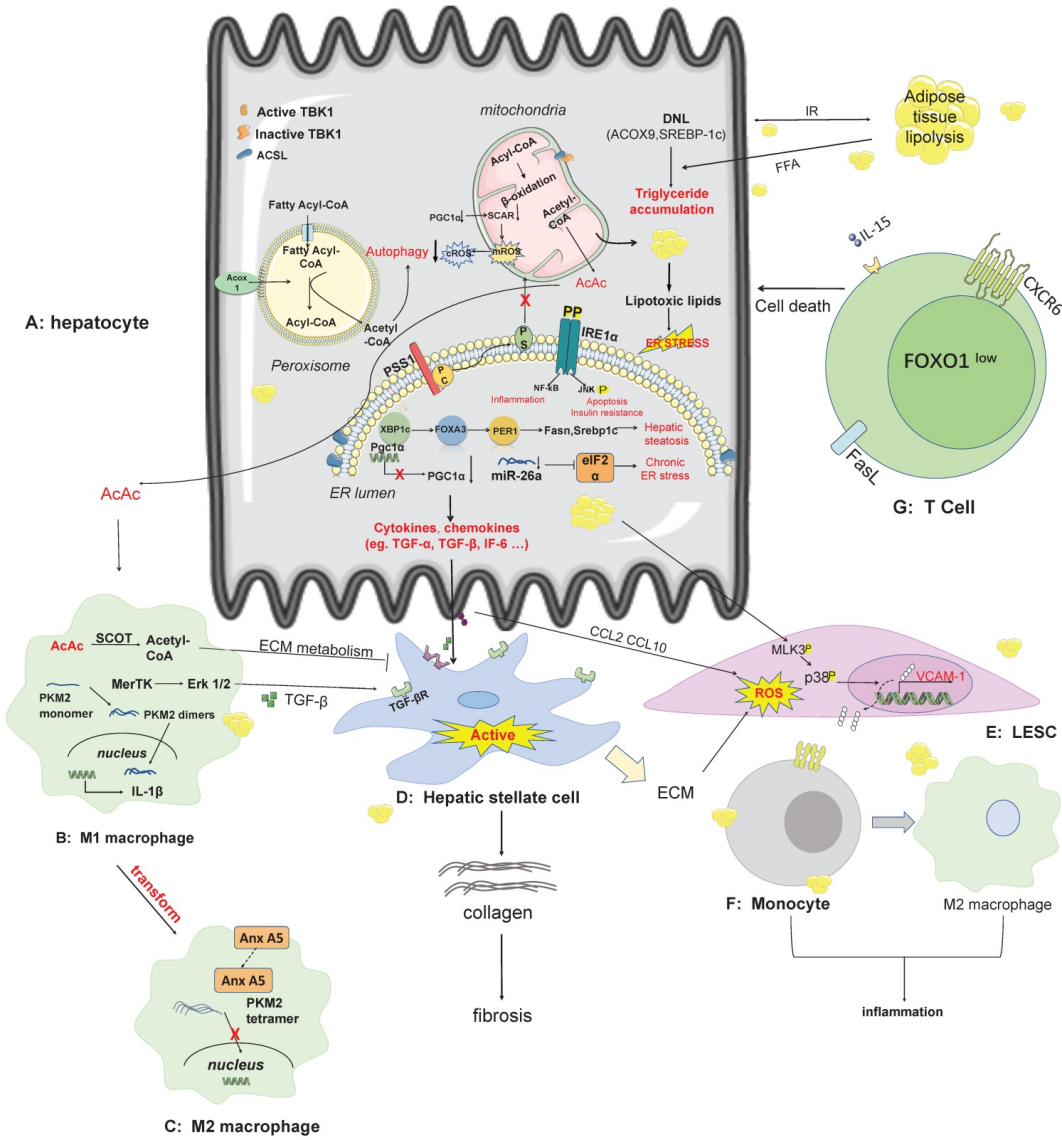
** The pathogenesis and therapeutic targets of NAFLD/NASH.** A: In hepatocytes, lipid synthesis, mitochondrial energy homeostasis, endoplasmic reticulum stress, novel mechanisms of peroxisomal involvement in the pathogenesis of NAFLD. The lipotoxicity arising from free fatty acid produced in hepatocytes elevates the secretion of cytokines and chemokines that affect other non-parenchymal cells, including kupffer cells, HSC, LESC. B-C: AcAc secreted by hepatocyte mitochondria can enter macrophages across cells, and metabolites of ECM can inhibit HSC activation when catalyzed by SCOT. Some pathways such as the MerTK-ERK1/2 pathway, produce TGF-β, which stimulates the activation of HSC. Anx A5 is a protein that influences the phenotypic switch from M1 to M2 in hepatic macrophages, which secrete cytokines with pro-inflammatory effects. D: HSC is stimulated by secreted proteins from other cell types and switches from a resting state to an activated state, producing collagen which in turn causes fibrosis. E-F: LSEC secretes VCAM-1 in a lipotoxic environment, and monocytes have receptors for VCAM-1 on their surface. Adhesion of LSEC to monocytes can contribute to the conversion of monocytes to M2 macrophages. G: CD8 T-cell production of auto-aggression involves IL-15-driven transcriptional programming. Self-attack by CD8 T cells in the liver is involved in liver injury via cell death pathways. Abbreviations: DNL: *de novo* lipogenesis; ECM: extra cellular matrix; IRE1a: nucleus signaling 1; LSEC: Liver sinusoids endothelial cell; PGC1a: PPAR-gamma co-activator-1 alpha; PSS1: phosphatidylserine synthase1; SCOT: succinyl-CoA:3-ketoacid CoA transferase; TBK1: TANK-binding kinase 1; VCAM1: vascular cell adhesion molecule-1.

**Table 1:** Comparison of mouse models of NAFLD/NASH

Animal model	Phenotype				Fibrosis	Advantage	Disadvantage	References
	IR	Obesity	Steatosis	Inflammation				
MCD	No	Weight loss	+++	+++	Yes	Short period; Easy to operate; High Reproducibility	No NAFLD-related metabolic syndrome	65, 159
CDAA	No	Weight loss	+++	++	Yes	Gain of weight; unclear increase in hepatic; peripheral insulin sensitivity	Long-period; high costs	160
High-fat diet (HFD)	Yes	Yes	+++	+	Yes	Low costs; easy to operate	Requires large sample size; difficult comparison between groups and protocols.	161
High-fructose diet	Yes	No	+++	++	No	Develop Metabolic syndrome.	It does not develop into advanced fibrosis or hepatocellular carcinoma; A high-fructose diet alone does not produce a Nash phenotype in the liver	162-164
High-cholesterol diet (HCD)	Yes	Yes	+++	++	Yes	Phenotype very similar to the clinical features of NASH in patients with metabolic syndrome	Not common in humans; Cholesterol in the diet may not be physiological	165, 166
WD+CCl_4_	No	No	+++	+++	Yes	Short-term; Progression to advanced liver disease; Simulating the histological, immunological and transcriptional characteristics of human NASH	IR was not observed in some models	95
HFD/HCD+CCl_4_	Yes	No	Yes	++	Yes			
STAM	Yes	No	+++	++	+	The whole process of NAFLD can be simulated with short time	Not include full range of human disease features	100
ob/ob	Yes	Yes (extremely)	+++	+	No	A number of features similar to human NAFLD appear	Rarely develop NASH without diet challenge	167, 168
db/db	Yes	Yes	+++	+	No	Same as above	Difficult to progress to advanced NASH	169
foz/foz	Yes	Yes	++	+	Yes	Phenotype very similar to the clinical features of NASH in patients with metabolic syndrome	Phenotype may be strain-dependent	170

**Abbreviations:** CDAA: Choline-deficient L-amino-defined diet; MCD: methionine- and choline-deficient diet; WD: Western diet;

**Table 2:** Established genetic targets of NAFLD/NASH: evidence from animal models and clinical trials

Gene	Animal model	Clinical trial number* and status
PNPLA3	HFD^171^-^174^	NCT04640324 (Completed, 2020)
		NCT01966627 (Completed, 2017)
		NCT02116192 (Completed, 2018)
TM6SF2	HFD 175	NCT04640324 (Completed, 2020)
	High-fructose diet model 176	NCT04501042 (Recruiting, 2020)
GCKR 2	HFD 177	NCT01966627 (Completed, 2017)
HSD17B13	Western Diet model 130	NCT04565717 (Recruiting, 2020)
	High-fat diet model 130	
TAZ	High-fat, high-glucose 178	/
	High-fat diet model 179	
	CDAHFD^180^	
	Genetically deficient mouse models^181^	

* https://clinicaltrials.gov

**Table 3:** Emerging pharmacotherapies for NAFLD/NAS

Target category	Drug name	Action	Animal model	Clinical trial number and status	Phase	Current Primary Outcome
Nuclear receptor	Pioglitazone	PPAR-γ agonist	HFD model^182^ CDAA diet model^183^	NCT03950505 (Recruiting, 2020)	4	In liver fibroscan, liver stiffness (kPa) as a marker of fibrosis and CAP (dB/m) as a marker of steatosis will be estimated.
				NCT03646292 (Recruiting, 2020)	3	More sensitive than the biopsy-based steatosis grade assessment in confirming liver fat changes.
	Elafibranor	PPARα/δ agonist	3D Spheroids^184^ Ob/Ob mice model^185^	NCT02704403 (Terminated, 2020)	3	Composite long-term outcome composed of all-cause mortality, cirrhosis, and liver-related clinical outcomes.
	Saroglitazar	PPARα/γ agonist	High fat western diet and ad lib sugar water (WDSW)^138^ High-Fat Emulsion/LPS Model^186^ HFHC model^187^	NCT03061721 (Completed, 2021)	2	Percentage change from baseline in serum ALT levels at Week 16.
				NCT03863574 (Completed, 2020)	3	NAS Score (NAFLD Activity Score)
	Obeticholic acid	FXR agonist	HFD model^47^ HFHC model^50^	NCT02548351 (Active, not recruiting, 2021)	3	To evaluate the effect of OCA on liver histology in non-cirrhotic NASH subjects with stage 2 or 3 fibrosis.
				NCT03439254 (Active, not recruiting, 2021)	3	Percentage of subjects with improvement in fibrosis by at least 1 stage with no worsening of NASH, using NASH Clinical Research Network (CRN) scoring system.
Incretin	Liraglutide	GLP 1 analogues	HFCC-CDX model^188^ Ob/Ob mice model^149^ HFD model^150^	NCT01237119 (Completed, 2016)	2,3	Liver histological improvement.
	Semaglutide	GLP 1 analogues	Diet-induced obesity (DIO) mice^189^ (40% fat, 40% carbohydrate and 2% cholesterol)	NCT03919929 (Recruiting, 2020)	2,3	Change in hepatic fat fraction.
				NCT04822181 (Recruiting, 2021)	3	Resolution of steatohepatitis and no worsening of liver fibrosis; Improvement in liver fibrosis and no worsening of steatohepatitis; Time to first liver-related clinical event (composite endpoint).
				NCT02970942 (Completed, 2020)	2	Percentage of Participants with Non- Alcoholic Steatohepatitis (NASH) Resolution Without Worsening of Fibrosis After 72 Weeks.
	Sitagliptin	DPP4 inhibitor	MCD model^190^ HFD model^191^ CDAA model^192^	NCT02263677 (Withdrawn, 2016)	4	Change in liver steatosis.
Anti-inflammatory and antioxidant effects	Alpha-lipoic acid	Nutraceutical	/	NCT04475276 (Not yet recruiting,2020)	4	Change in fatty liver grading in NAFLD assessed by abdominal ultrasound.
Immunomodulator	Selonsertib^175^	ASK1 inhibitor	/	NCT03053063 (Terminated, 2020)	3	Percentage of Participants Who Achieve a ≥ 1-Stage Improvement in Fibrosis According to the NASH Clinical Research Network (CRN) Classification Without Worsening of NASH.
Inflammation	Cenicriviroc	CCR2/5 antagonist	HFHF model^155^	NCT03028740 (Terminated, 2021)	3	Superiority of CVC compared to placebo on liver histology at Month 12 relative to the Screening biopsy; Superiority of CVC compared to placebo on the composite endpoint of histopathologic progression to cirrhosis, liver-related clinical outcomes, and all-cause mortality.

## Abbreviations

ABCG5: ATP-binding cassette sub-family G member 5
ASK1: apoptosis signal-related kinase 1
ATGL: adipose triglyceride lipase
CDAA: choline-deficient L-amino-defined diet
CDHFD: choline-deficient HFD
CVC: cenicriviroc
DCGI: Drug Control Center of India
DMN: dimethylnitrosamine
EBP: emopamil binding protein
ELA: elafibranor
FXR: farnesol X receptor
GVB: gut vascular barrier
GWAS: genome-wide association study
HFD: high-fat diet
HFE: high-fat emulsion
HFFD: high-fat and high-fructose diet
HFHC: high-fat and high-cholesterol diet
hiPSC: human induced pluripotent stem cells
HLF: human lung fibroblasts
HSC: hepatic stellate cell
HTGC: hepatic triglyceride content
IRS2: insulin receptor substrate 2
JNK: c-Jun N-terminal kinase
LDs: lipid droplets
LPS: lipopolysaccharides
LSEC: liver sinusoidal endothelial cells
MCD: methionine- and choline-deficient diet
MC4R-KO: melanocortin 4 receptor-deficient
NAFLD: non-alcoholic fatty liver disease
NASH: non-alcoholic steatohepatitis
OCA: obeticholic acid
PNPLA3: patatin-like phospholipase domain 3
PPARs: peroxisome proliferator-activated receptors
RA: rosemary acid
SAR: saroglitazar
SEL: selonsertib
TLR4: toll-like receptor 4
WD: western diet
WDF: WD supplemented with fructose
3D-hLMT: three-dimensional (3D) human liver microtissue
